# Supplementary material for: Implementing a physical activity project for people with dementia in Germany–Identification of barriers and facilitator using consolidated framework for implementation research (CFIR): A qualitative study
Source: PLoS One. 2023 Aug 9;18(8):e0289737. doi: 10.1371/journal.pone.0289737 (PMC10411781; doi:10.1371/journal.pone.0289737)
Supplement: S1 Table — (PDF) [file pone.0289737.s001.pdf]

SEMI-STRUCTURE INTERVIEW GUIDE	
Verbal informed consent	
**CFIR DOMAIN & CONSTRUCT**	**QUESTION**
**I. Intervention characteristics**	
Relative Advantage	What were the advantages of implementing this project/sport initiative on your setting?
Adaptability	To what extent the project/sport initiative was adequate to meet specific local needs?<br>What kinds of changes or alterations did you have to make to the project/sport initiative so it worked effectively in your setting?
Intervention Source	Why the project/sport initiative was implemented in your setting?<br>What is your perception of whether the project/sport initiative development originated from external or internal sources?
Complexity (barriers)	How complicated was to implement the project /sport initiative?
**II. Characteristics of Individuals**	
Motivation	What was your level of motivation to implement the project/sport initiative? Why?
Knowledge and beliefs about the intervention	How did you feel about implementing the project/sport initiative for PwD in your setting?
Self-efficacy	In a scale from 1 to 5, how confident were you to be able to successfully implement the project/sport initiative? When 1 = "No confident at all" and 5 = "very high confident". Why?
**III. Process**	
Planning	What have you done to get a plan in place to implement the project/sport initiative?
Engaging - Formally appointed internal implementation leaders	Who led implementation of the project/sport initiative?<br>What attributes or qualities did this person have that made them an effective leader of this implementation?
Executing	Has the project/sport initiative been implemented according to the implementation plan?<br>[If Yes] Can you describe this?<br>[If No] Why not?
Reflecting & Evaluating	How did you assess progress towards implementation or project/sport initiative goals?
**IV. Inner Setting**	
Implementation Climate	What was the general level of receptivity in your organization to implementing the project/sport initiative? Why?
Compatibility	How well did the project/sport initiative fit with existing work processes and practices in your setting?

Learning Climate	To what extent do you feel like you can try new things to improve your work processes, express your needs for assistance, and to find time for discussion and evaluation within your sports association?
Relative Priority	What was the priority of getting the project/sport initiative implemented relative to other initiatives that were happening in your sports association?
Communication	Did PLs and STs meet formally or informally? How often did you meet? Formally? Informally?
Available resources	Were there available resources to help ensure that the implementation of the project/sport initiative?
**V. Outer Setting**	
Cosmopolitanism	To what extent did you network with colleagues or people in similar professions/positions outside your setting?
External Policies & Incentives	What kind of local, state, or national performance measures, policies, regulations, or guidelines influenced the decision to implement the project/sport initiative?
